# Heated Relations: Temperature-Mediated Shifts in Consumption across Trophic Levels

**DOI:** 10.1371/journal.pone.0095046

**Published:** 2014-05-05

**Authors:** Linda I. Seifert, Francisco de Castro, Arnim Marquart, Ursula Gaedke, Guntram Weithoff, Matthijs Vos

**Affiliations:** 1 Department of Ecology and Ecosystem modelling, Potsdam University, Potsdam, Germany; 2 School of Biological Science, Queen's University of Belfast, Belfast, United Kingdom; 3 Berlin-Brandenburg Institute of Advanced Biodiversity Research (BBIB), Berlin, Germany; 4 Institute of Environmental Sciences (CML), Leiden University, Leiden, the Netherlands; CSIR- National institute of oceanography, India

## Abstract

A rise in temperature will intensify the feeding links involving ectotherms in food webs. However, it is unclear how the effects will quantitatively differ between the plant-herbivore and herbivore-carnivore interface. To test how warming could differentially affect rates of herbivory and carnivory, we studied trophic interaction strength in a food chain comprised of green algae, herbivorous rotifers and carnivorous rotifers at 10, 15, 20 and 25°C. We found significant warming-induced changes in feeding by both herbivorous and carnivorous rotifers, but these responses occurred at different parts of the entire temperature gradient. The strongest response of the per capita herbivore's ingestion rate occurred due to an increase in temperature from 15 to 20°C (1.9 fold: from 834 to 1611 algal cells per h^−1^) and of the per capita carnivore's ingestion rate from 20 to 25°C (1.6 fold: from 1.5 to 2.5 prey h^−1^). Handling time, an important component of a consumer's functional response, significantly decreased from 15 to 20°C in herbivorous rotifers. In contrast, it decreased from 20 to 25°C in carnivorous rotifers. Attack rates significantly and strongly increased from 10 to 25°C in the herbivorous animals, but not at all in the carnivores. Our results exemplify how the relative forces of top-down control exerted by herbivores and carnivores may strongly shift under global warming. But warming, and its magnitude, are not the only issue: If our results would prove to be representative, shifts in ectotherm interactions will quantitatively differ when a 5°C increase starts out from a low, intermediate or high initial temperature. This would imply that warming could have different effects on the relative forces of carnivory and herbivory in habitats differing in average temperature, as would exist at different altitudes and latitudes.

## Introduction

Global change will most likely result not only in higher mean temperatures, but also in more variable ones in temperate regions [1], and is projected to significantly affect species interactions in the pelagic communities of lakes and oceans in the coming century [2], [3], [4], [5]. Such warming may cause (i) temporal mismatches between predators and prey, (ii) reduced body sizes, (iii) species range shifts, (iv) extended durations of stratification and (v) large scale changes in the spatial distribution of primary and secondary production e.g. [6], [7], [8], [9], [10].

While warming itself is a slow, gradual process, forecasts also predict an increased frequency of extreme events, such as heat waves. These act at the time scale of days and will affect individual organisms within their lifetime [11]. The ensuing individual responses and changes in species interactions are likely to have short-term cascading effects at the community level. Effects on terrestrial and aquatic communities could be similar (especially for insects and zooplankton), although water acts as a buffer that delays temperature changes.

Warming will likely accelerate all physiological processes, but not all at the same rate for all organisms [12], [13], [14] , trophic levels [15] or locations [16]. A rise in temperature may generally increase movement rates of ectotherms and intensify the feeding links involving these ectotherms in aquatic food webs. However, the effects could differ between the plant-herbivore interface and the herbivore-carnivore interface. Mobile herbivorous zooplankton species attack relatively immobile phytoplankton. Flagellated phytoplankton species exist, but their movement rate is negligible in comparison to that of their much larger zooplankton consumers. In contrast, many mobile carnivore species attack mobile herbivores. Preliminary (unpublished) modelling by M. Vos showed that a mobile predator searching for mobile individuals on a two-dimensional grid experienced a higher encounter rate than one searching for stationary individuals. This led to the idea that warming could increase encounter rates between mobile carnivores and mobile herbivores more strongly than the encounter rate between mobile herbivores and relatively immobile phytoplankton. Recently, an analysis of asymmetries in the thermal responses of a large number of consumer-resource systems by [17] indicated that these are likely to commonly occur in nature. How increased encounter rates ultimately change actual predation rates requires careful study, as warming may also increase escape velocities of prey attacked by faster predators [18],[19].

When increased rates of warming lead to an intensification of species interactions, this could cause stronger population fluctuations, rapid local loss of carnivores and accelerated rates of competitive exclusion among herbivores [20], [21]. In summary, it could change the importance of top-down and bottom-up effects [22] and modify trophic cascades [23]. Thus, it may lead to community instability, and, indeed, much of the available evidence for ectotherms points in this direction, both in theory [20] and in experimental studies [24], [25], [26], [27], [28], [29]. Such destabilisation could in turn cause cascading extinctions and community closure [30], with profound implications for the persistence and conservation of these systems. It is important to gain more insight into the underlying mechanisms and into its generality, as some theoretical work has actually predicted the opposite effect, a stabilization of dynamics [19].

Part of the instability of many predator-prey systems arises from a type-II functional response of the predator, that destabilizes dynamics compared to type-I and type-III functional responses [31], [32], [33]. However, a type-III functional response should not be seen as a guarantee for stability. For example, the predator *Stenostonum* has a type-III functional response [34] but it overexploits its prey to extinction within a couple of days in the laboratory. As warming may affect the behavioural parameters that determine any ectotherm's ingestion rate, it is important to study how warming affects the functional response and thus the feeding link intensity and propensity to population level instability. In general, one would expect a higher activity level at higher temperatures, implying higher attack rates and lower handling times. However, a wide range of alternative patterns have been described: shifts between functional response types; attack rates and handling time may increase, decrease or stay the same, and the observed change may be gradual or discontinuous across a range of temperatures [35], [36], [37], [38], [39], [40], [41], . The relationship between search rate and temperature has for instance been reported to be linear, quadratic or hump-shaped [35], [36], [37], [38], [39], [40], [41], . Part of this variation is likely to stem from the fact that responses to a higher temperature are not only expected to increase indefinitely, but to actually drop again once a threshold temperature has been reached. [43] describe the theoretically expected hump-shape of temperature response curves for ectotherms and give some empirical examples, also see [44], [45], [46].

In order to test our ideas on the effects of warming on carnivory and herbivory we use a specific well-studied model system, and formulate the following hypotheses: (1) Ectothermic herbivores and carnivores will show an increase in consumption rates under warming. (2) The extent of the effect, and the temperature range for which it occurs, will differ between consumers at different trophic levels. The underlying consideration is that the mode of how food is taken in may often differ between herbivores and carnivores. Focusing on a zooplankton example, it is clear that foraging for immobile tiny algal particles is a fundamentally different process, with different costs and benefits, than foraging for mobile and much larger animal prey items. Our model system is a specific one, but this difference in mobility of plant and animal prey also holds for a large number of other aquatic and terrestrial ectotherms. (3) The effect of warming on ingestion is not necessarily gradual and linear. The effect of warming from low to intermediate temperatures is not necessarily the same as it is from intermediate to high temperatures. To test our hypotheses, we studied herbivore and carnivore ingestion rates, in our planktonic model system, in relation to food concentration along a temperature gradient formed by 5°C steps from 10°C to 25°C. A response to a 5°C increase in temperature is ecologically relevant as it could easily occur within the lifetime of an individual rotifer experiencing a heat wave.

## Materials and Methods

### Culture organisms

All organisms were cultured in modified Woodshole (WC) medium [47] medium. These included the alga *Monoraphidium minutum* (SAG 243-1, culture collection of algae, Göttingen, Germany), the herbivorous rotifer *Brachionus calyciflorus* (isolated from Lake Michigan, Milwaukee, USA; obtained from G. Fussmann, McGill University, Montreal, Canada) and the carnivorous rotifer *Asplanchna brightwellii* (isolated from Lake Ismaningen, Munich, Germany; obtained from C. Laforsch, Ludwig-Maximillian University Munich, Germany). Stock cultures were kept as batch cultures at 20°C with regular substitutions of fresh medium.

An amount of 1000 algal cells of *M. minutum* correspond to a content of 0.009 µg C at 20°C and one individual of *B. calyciflorus* contains of about 0.05 µg C at 20°C.

### Herbivory

We tested *B. calyciflorus* grazing on *M. minutum* at different temperatures and prey densities. All organisms experienced an acclimation period of 24 hours to the experimental temperatures (10, 15, 20 and 25°C). Rotifers were acclimated in an algal suspension of 3×10^5^cells/ml to ensure good physiological conditions at the beginning of the experiment. After acclimation, 20 full-grown rotifers without eggs were selected and transferred into 3-ml glass flasks of five different algal concentrations (0.2, 1, 3, 6 and 10×10^5^ cells/ml). Twenty animals per 3 ml is much below the critical consumer density where consumption decreases due to crowding effects [48]. Grazing occurred in the dark at 10, 15, 20 and 25°C in climate-controlled chambers (Minitron INFORS HT, Bottmingen, Switzerland) for 24 hours. This period was sufficient to observe measurable grazing. Two algal samples (‘start’ and ‘end’ sample) without rotifers served as controls. Algal ‘start’ samples were immediately fixed (by adding Lugol's iodine solution) at the beginning and algal ‘end’ samples were terminated after 24 h, to calculate algal growth or decline during the experiment. The grazing samples with rotifers were fixed after 24 h as well. For each temperature treatment and food concentration, experiments were run with 6 replicates and 4 controls. Algal densities were determined with an inverted microscope using the cell counting method [49]. At least 600 algal cells were counted for each sample. In order to estimate algal food density during the experiment, food concentrations were integrated to correct for algal depletion. In our results we use these corrected food densities rather than the experimentally implemented initial food densities. The ingestion rate (*I*) was calculated on basis of the filtration rate per animal and time (*f*) and the algal concentration (*C*). Filtration was calculated as:
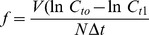
where *V* is the experimental volume and *C_t0_* and *C_t1_*are the algal densities at the beginning and the end of the experiment, respectively. N is the number of animals and *Δt* is the duration of the experiment. Ingestion rate (*I*) is then f multiplied by algal density.

### Carnivory

*A. brightwellii* and its prey, *B. calyciflorus*, were acclimated to the experimental temperatures (10, 15, 20 and 25°C) for 24 h. Prior to the experiment, full-grown predators were subjected to three hours of starvation. One *A. brightwellii* individual was transferred into each of the experimental 1.5 ml-wells with different prey densities (2, 5, 7, 10, 12, 15, 17, 20, 25 and 30 individuals/ml). Predation occurred at 10, 15, 20 and 25°C in the aforementioned Minitrons, for 3 hours. Treatments without carnivores served as controls. A number between 6 and 15 replicates and 4 controls were used for each temperature and food concentration. The experiment was terminated by adding Lugol's iodine solution and the entire volume was screened using a dissecting microscope for remaining prey individuals. Ingestion rate was calculated as the difference between the number of remaining prey and the mean number of prey in the controls.

### Curve fitting of functional responses

Visual inspection of the data suggested that either a type-II or a type-III functional response could be an appropriate model, and we fitted both of these to the data. We also fitted integrated functional response models to address the issue of prey depletion during the experiment (we could not replace algae or rotifers at the time they were eaten) [50]. For completeness, we additionally fitted a linear Holling type-I functional response with a plateau (a ‘broken-stick model’). In this model, the ingestion rate increases linearly with prey density up to a certain point (which is estimated during the fitting), after which it remains constant. The fitting was done in Matlab (v 7.0) by non-linear least squares, with the minimum values of all parameters bounded to 0. Maximum values were unbounded for attack rate and handling time.

Instantaneous functional response:

The generalized form of the Holling functional response is
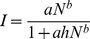
(1)where *I* is ingestion, *N* is prey abundance, *a* denotes attack rate, *h* stands for handling time and *b* is the Hill exponent. Changing the value of *b* makes the model to go from type-II (*b* = 1) to type-III (*b* = 2).

Integrated functional response:

The integrated generalized functional response results from integrating (see Table S1 in Appendix S1), between time 0 and *T*, the model:
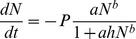
(2)which results in:
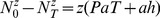
(3)where N_0_ and N_T_ are the initial and final prey densities, respectively, *z* is equal to *b*+1, *P* is the number of predators and *T* is the duration of the experiment (*a* and *h* as in model (1)). In the particular case of Holling type-II (*b* = 1), the integrated form of the functional response is:
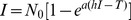
(4)For Holling type-III (b = 2), the solution is:
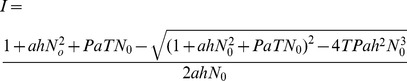
(5)Incidentally, in [51] this equation is incorrect. Equations (3) and (4) are implicit functions, which cannot be fitted by conventional methods. Thus we fitted them in the form:
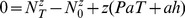
(6)for model [3], and:
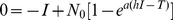
(7)for model (4).

To quantify the forces of top-down control by herbivorous and carnivorous rotifers at different temperatures, we calculated ‘fold-increases’ in ingestion rate using the fitted functional response at high food density (1 10^6^ cells/ml and 30 prey/ml, respectively)

## Results

### Functional responses at different temperatures, curve fitting

As expected from visual inspection, both type-II and type-III functional response models provided a good fit to the herbivory and carnivory ingestion data. The adjusted R^2^ values (i.e. corrected for the number of parameters) showed only marginal differences in goodness of fit, with more of the values for the different temperature treatments indicating a slightly better fit of a type-III model, both for herbivory and for carnivory with R^2^ ranging from 0.35 to 0.73 and from 0.14 to 0.33, respectively (see Table S1 in Appendix S1).

Therefore we accepted, with caution and taking notice of the considerable scatter in the data, the type-III model as a good description of the functional responses. Given that our data were based upon an experiment in which depletion occurred, we based our subsequent analyses on the integrated type-III functional response model. This model is the most appropriate given the method used and shows a goodness of fit that is nearly identical to that for the non-integrated type-III model (Table S1 in Appendix S1).

The broken-stick (type-I) model generally reached lower adjusted R^2^ values than the Holling type-II and -III models and will hence not be considered any further.

### Herbivory

**Figure 1:**
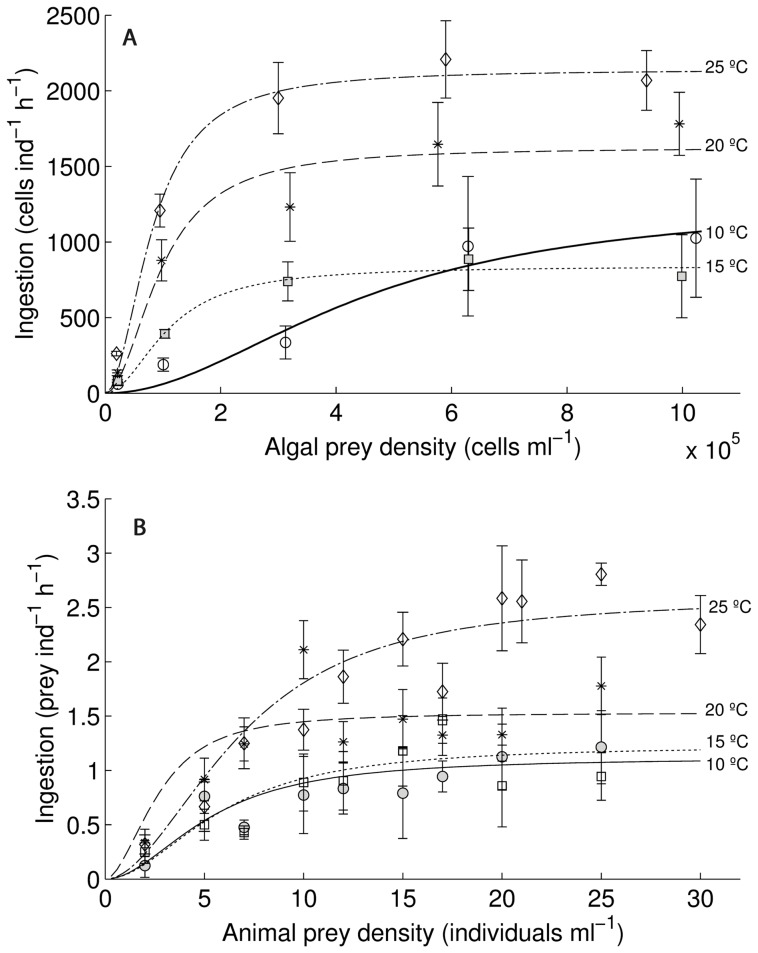
Ingestion, with fits of the integrated Holling type-III functional response to the data for (a) herbivory and (b) carnivory, shown as lines. Dots and bars represent means and standard errors. Circles and thick continuous line: 10°C; squares and dotted line: 15°C; stars and dashed line: 20°C; Diamonds and dot-dash line: 25°C.

Ingestion rates by herbivorous rotifers differed with temperature and food concentration. Functional response curves were most clearly separated between different temperatures at higher food concentrations (Fig. 1A). At 10°C no saturation of the ingestion rate was observed for the tested range of food levels.

At lower food concentrations ingestion rates gradually increased with temperature. Herbivore ingestion rates were more similar between 10°C and 15°C on the one hand and between 20 and 25°C on the other hand, with some distance between these groups. For this reason, at high food densities the greatest change in ingestion out of all of the 5°C steps occurred between 15 and 20°C. This increase resulted in an increase of the functional response plateau (maximal ingestion rate) of about 800 cells ind^−1^ h^−1^. Hence, from low temperatures to high temperatures ingestion by individual herbivorous consumers increased by factors of about 1.9 and 2.6 from 15 to 20 and 25°C, respectively.

**Figure 2:**
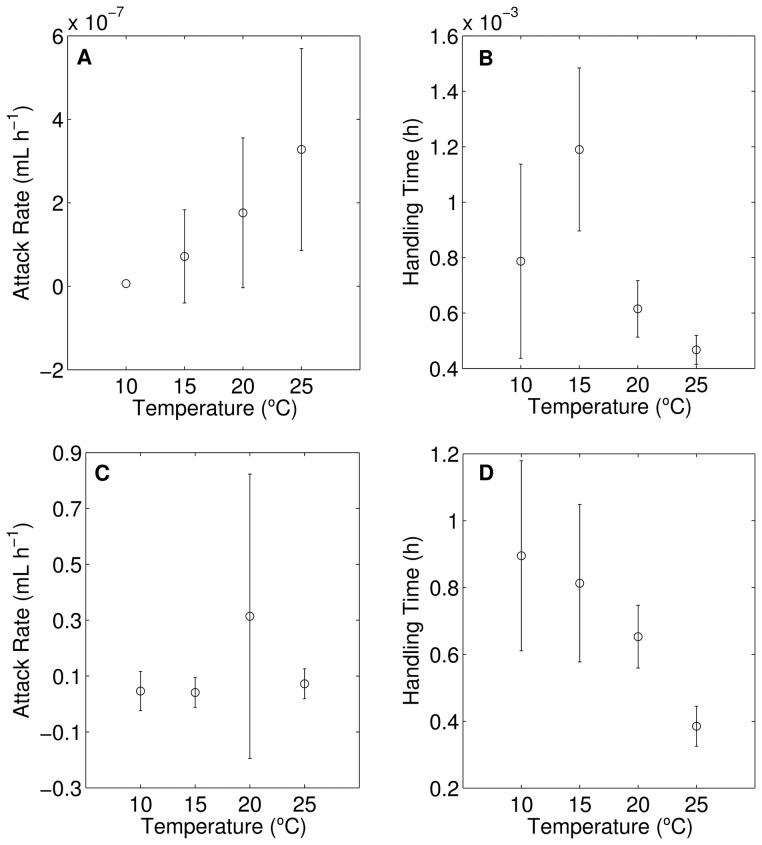
Functional response parameters estimated for the integrated type-III model for herbivore per capita attack rate (a) and handling time (b) and carnivore per capita attack rate (c) and handling time (d) at each temperature (units are given on the ordinates). Bars represent the upper and lower 95% confidence limits. In the fitting, the handling time at 10°C for the herbivore reached its lower boundary (zero), thus confidence intervals were not estimated (panel a). At 10°C the herbivore's functional response did not reach a plateau, for which reason the estimated handling time at this temperature is less reliable than the others (panel b).

Functional response parameters from the integrated type-III model provided clear quantification of the observed differences in functional response shape with temperature. Attack rates of herbivores gradually increased with temperature showing a significant difference between 10 and 25°C, indicated by non-overlapping confidence intervals for these temperatures (Fig. 2A). Mean values of the per capita attack rate increased as: 0.06×10^−7^, 0.72×10^−7^, 1.76×10^−7^ and 3.28×10^−7^ mL h^−1^ for 10, 15, 20 and 25°C, respectively. Herbivore handling time significantly decreased from 1.19×10^−3^ to 0.62×10^−3^ h from 15 to 20°C, but did not significantly change between 20 and 25°C, remaining at about 0.47×10^−3^ h (Fig. 2B). As a plateau for ingestion rate was absent at 10°C for the tested range of food densities we could not meaningfully compare the handling time at this temperature with the handling times at the three higher temperatures (noting that 1/handling time defines the plateau).

### Carnivory

Ingestion rates of the carnivore differed with temperature and prey concentration. As for herbivores, functional responses were most clearly separated between temperatures at higher food concentrations (Fig. 1B). The slope of increase in ingestion rate was similar for the 10 and 15°C data, but separated with further increases in temperature. From 20 to 25°C maximum ingestion rate significantly increased, by a factor of 1.6.

As for herbivory, the differences in shape of functional responses by carnivores were most clearly quantified by the estimated attack rate and handling time. Estimated attack rates for the carnivore did not significantly change along the entire temperature gradient from 10 to 25°C (Fig. 2C). Handling times by the carnivore on its herbivorous prey did not significantly decrease along the range from 10 to 20°C, but showed a significant drop between 20 and 25°C (Fig. 2D).

## Discussion

If global change proceeds as projected, individuals in ectotherm communities will experience higher and more variable temperatures, including environmental extremes such as heat waves. The ecological consequences of those changes could be profound. Warming-induced intensification of species interactions could in theory lead to stronger population fluctuations and as a consequence, to a loss of top-predators [20]. Consequently, communities could be destabilized, and this could in turn lead to cascading extinctions and community closure [30]. Ectothermic animals comprise the majority of all multicellular animal species on the planet and, hence, it is important to study how different types of ectothermic species respond to warming. One important difference in response might occur between carnivores and herbivores. Such a difference could lead to changes in the degree of top-down control between the plant-herbivore and herbivore-carnivore interface and at different parts of the entire temperature gradient.

We studied experimentally ingestion rates of herbivorous and carnivorous rotifers in relation to food concentration along a temperature gradient from 10 to 25°C, in 5°C steps. These rotifer species provide a realistic example, as they co-occur in natural planktonic food chains. We thus tested whether temperature-induced effects differed between trophic levels and between different temperature steps (along different parts of the entire temperature gradient). We quantified differences in feeding responses along the temperature gradient on the basis of fits of functional response models to the data. Here we discuss differences for herbivores and carnivores on the basis of ingestion rates and the estimated functional response parameters attack rate and handling time.

### Feeding across trophic levels

We found significant warming-induced changes in the feeding of both herbivorous and carnivorous rotifers. Overall, ingestion rates increased with warming, the largest separation between herbivore functional response curves occurred as temperature increased from 15 to 20°C, while for the carnivore this occurred between 20 and 25°C. Responses to warming thus differed between these different kinds of consumers, as the ‘major jumps’ in feeding occurred at different parts of the temperature gradient. To quantify this finding more precisely, we discuss it further below in terms of differences between the functional response parameters handling time and attack rate that actually define the shape of these curves.

### Handling time

The observed differences between functional response curves could largely be attributed to differences in handling time. Feeding rate was then most likely determined by the integrated effect of both actual handling time and gut fullness. Actual observed handling times of carnivorous rotifers on herbivorous rotifers span the range from seconds on undefended small individuals to about 20 minutes for a fully defended spined herbivorous rotifer (*B. calyciflorus*, Seifert, personal observation). A plateau of between 1 and 2.5 rotifer prey per hour as seen in Figure 1 is thus likely not due to actual handling time alone.

### Attack rate

Herbivore attack rates showed a gradual increase from 10 to 25°C, with only the difference between these extremes being significant. Attack rate determines the functional response shape at low food densities. Therefore the observed response to warming will be most relevant to environments where prey densities are low. Mean attack rates of herbivores continuously increased which, however, was only significant through an increase in ambient temperature of 15°C. Carnivore attack rates did not significantly increase from 10 to 25°C. We thus observed differences in attack rate responses to warming between the herbivore and carnivore. However, these were not responsible for the main differences among ingestion rates.

Prior to performing the experiment we hypothesized that warming would increase the rate of herbivory by increasing encounter rates between mobile herbivores and their immobile prey. However, we only found a significant increase in attack rates under a rather extreme increase in temperature of about 15°C (from 10 to 25°C). The main response explaining increased herbivore ingestion rates was a significantly reduced handling time, with the drop happening between 15 and 20°C. It was therefore not the food encounter rate, but the food processing rate that increased, and led to an increase in the force of herbivory. So, we originally phrased our hypothesis in terms of temperature effects on movement, as discussed in [17] but we found that, for this particular model system, food processing rates were of overriding importance when compared to effects on body velocity. We consider it likely that gut evacuation times are shorter at 20°C than at 15°C, which allows the herbivores to have a higher maximum ingestion rate.

### Warming effects on mobility or on food processing rates

Rates of carnivory are determined by the mobility of both carnivores and their herbivorous prey. Warming could lead to higher encounter rates between them, as both tend to move more quickly at higher temperatures. However, prey escape ability might also be enhanced under warming [19]. It was therefore not straightforward what the exact end-result of warming would be for realized rates of carnivory.

What we observed is that actual encounter rates as measured by carnivore attack rate did not show a significant increase over the entire temperature gradient. This is in contrast with the pattern observed for herbivores, where such an increase clearly occurred. So, for carnivores a potential increase in encounter rates may have been counterbalanced by an increase in successful escapes by herbivorous prey.

However, like in herbivores, the main effect on carnivory occurred through an increase in prey processing rate, i.e. by a reduction in handling time. However, as stated above, this happened at another part of the temperature gradient, under an increase from 20 to 25°C. Our observations on this model system raise the question whether ectotherm carnivores in general increase their food processing rates at higher temperatures than ectotherm herbivores. Future research needs to address this question in more detail, probably in relation to additional explanatory variables such as body size, stoichiometry type of movement and the altitude/local climatic context of the interaction.

### Functional response types

The difference in how well different functional response types fitted our data was small, which is understandable given the variation in the data. We have therefore chosen to emphasize the major patterns, in terms of the clearly different levels of predation at different temperatures, rather than to focus on differences in functional response type. Both type-II and type-III functional responses have ranges of prey density for which the individual prey risk of being consumed is decreasing with prey density [33] and hence the interaction between predator and prey is destabilized [31]. That instability is possible and increases under warming is evident from most of the experimental studies of population dynamics in ectotherms [24], [25], [26], [27], [28], [29].

### Potential consequences at the food chain level

We studied a relatively wide range of temperatures, from 10 to 25°C. The animals were feeding at all the tested temperatures, suggesting we did not cross the boundaries of the thermal windows for the tested herbivorous and carnivorous rotifers. No clear effect on top-down control emerged for warming from 10 to 15°C, as neither handling time, nor attack rate showed a significant change in either the herbivore or the carnivore under such warming. However, when a 5°C increase in temperature started from an initial temperature of 15°C, top-down control at the herbivore-plant interface increased, whereas the force of carnivory stayed the same. When warming lead to a further increase in temperature, from 20 to 25°C, no further change in the force of herbivory took place. The increase in the force of top-down control then occurred at the carnivore-herbivore interface. We quantified the above pattern by calculating the ‘fold-increase’ ingestion rate for a given 5°C increase in temperature for saturating consumption (i.e. at a high prey density). These calculations revealed a 1.9-fold increase in the herbivore's ingestion rate for an increase in temperature from 15 to 20°C (from 834 to 1611 algal cells per ind^−1^ h^−1^) and a 1.6-fold increase in the carnivore's ingestion rate for an increase in temperature from 20 to 25°C (from 1.5 to 2.5 prey ind^−1^ h^−1^). Quantitatively showing the consequences for (in-)stability of the entire food chain and the ensuing population fluctuations (and resulting local extinction risks) are beyond the scope of this paper, but will be evaluated in a separate study.

Models predicted either destabilization [20] or stabilization [19], [52] of food chains under warming depending on which part of the thermal window of the component organisms was considered. Destabilization is expected when warming intensifies the trophic interaction whereas a stabilizing effect may occur when temperatures approach the upper limit of the thermal window, where an imbalance between respiration rate and food intake leads to reduced feeding rates. [53] determined the maximum oxygen consumption rate of *B. calyciflorus* to occur at about 32–33°C and the growth rate in this species showed a sharp decrease at 39°C when compared to growth at 35 and 37°C, most likely because increased consumption can no longer compensate for the strong temperature-induced increase in respiration rate. As this point was only reached at a much higher temperature than the warming gradient tested in our study (5 to 25°C), we consider it unlikely that increases in respiration ‘outran’ the observed increases in ingestion rates of our experimental animals. Hence, we hypothesize that moderate warming of this ectotherm food chain considered in our study will, in a temperate system, result in an intensification of consumption that most likely leads to a destabilization of predator-prey interactions, both at the plant-herbivore and at the herbivore-carnivore interface. In a warmer climate, or one in which more heat waves occur, ectotherms will spend more time at higher temperatures each summer than with the current climate, leading to prolonged periods during which interactions are destabilized [54]. for example reported quantitative details of the consequences of the 2003 European heat wave, for temperature profiles of a range of European lakes. For our specific system, we observed differences in the warming-induced increases in the force of herbivory and carnivory along the temperature gradient. Such differences could translate into quantitative variation in the exact degree of destabilization across temperature gradients.

Our results exemplify how the relative forces of top-down control exerted by herbivores and carnivores may strongly shift under global warming. If our results prove to be representative, changes in ectotherm interactions will quantitatively differ when a 5°C increase starts out from a low, intermediate or high initial temperature which could have far reaching consequences for food web functioning in habitats with different thermal regimes, see [55]. Further research concerning warming effects at the community level is needed to predict consequences for natural ecosystems and to contribute to management strategies maintaining ecosystem services.

## Supporting Information

Appendix S1 (DOCX)

## References

[1] Intergovernmental Panel on Climate Change (2007) Climate Change 2007: Synthesis Report.

[2] Levitus S, Antonov JI, Boyer TP, Stephens C (2000) Warming of the world ocean. Science 287: 2225–2229.

[3] Hansen J, Sato M, Ruedy R, Lo K, Lea DW, et al (2006) Global temperature change. PNAS 103: 14288–14293. 17001018 10.1073/pnas.0606291103 PMC1576294

[4] Wagner A, Benndorf J (2007) Climate-driven warming during spring destabilizes a *Daphnia* population: a mechanistic food web approach. Oecologia 151: 351–364. 17120058 10.1007/s00442-006-0554-5

[5] Richardson AJ (2008) In hot water: zooplankton and climate change. Ices J Mar Sci 65: 279–295.

[6] Edwards M, Richardson AJ (2004) The impact of climate change on the phenology of the plankton community and trophic mismatch. Nature 430: 881–884. 15318219 10.1038/nature02808

[7] Richardson AJ, Schoeman DS (2004) Climate impact on plankton ecosystems in the Northeast Atlantic. Science 305: 1609–1612. 15361622 10.1126/science.1100958

[8] Winder M, Schindler DE (2004) Climatic effects on the phenology of lake processes. Glob Change Biol 10: 1844–282.

[9] Visser ME, Both C (2005) Shifts in phenology due to global climate change: the need for a yardstick. Proc R Soc 272: 2561–2569. 10.1098/rspb.2005.3356 PMC1559974 16321776

[10] Daufresne M, Lengfellner K, Sommer U (2009) Global warming benefits the small in aquatic ecosystems. PNAS 106: 12788–12793. 19620720 10.1073/pnas.0902080106 PMC2722360

[11] Sentis A, Hemptinne JL, Brodeur J (2013) Effects of simulated heat waves on an experimental plant-herbivore-predator food chain. Glob Change Biol 19: 833–842. 10.1111/gcb.12094 23504840

[12] Bělehrádek J (1957) Physiological aspects of heat and cold. Annu Rev Physiol 19: 59–82. 13412051 10.1146/annurev.ph.19.030157.000423

[13] Wieser W (1973) Effects of temperature on ectothermic organisms: Ecological implications and mechanisms of compensation. Springer Berlin Heidelberg

[14] Rombough P (2003) Modelling developmental time and temperature. Nature 424: 268–269. 12867969 10.1038/424268a

[15] Voigt W, Perner J, Davis AJ, Eggers T, Schumacher J (2003) Trophic levels are differentially sensitive to climate. Ecology 84: 2444–2453.

[16] Huston MA (2003) Heat and biodiversity. Science 299: 512–513. 12546005 10.1126/science.299.5606.512

[17] Dell AI, Pawar S, Savage VM (2014) Temperature dependence of trophic interactions driven by asymmentry of species responses and foraging strategy. J Anim Ecol 83: 70–84. 23692182 10.1111/1365-2656.12081

[18] Smolinský R, Gvoždík L (2013) Effect of temperature extremes on the spatial dynamics of predator-prey interactions: a case study with dragonfly nymphs and newt larvae. J Thermal Biol 39: 12–16.

[19] Vucic-Pestic O, Ehnes RB, Rall BC, Brose U (2011) Warming up the system: higher predator feeding rates but lower energetic efficiencies. Glob Change Biol 17: 1301–1310.

[20] Vasseur DA, McCann KS (2005) A mechanistic approach for modeling temperature-dependent consumer-resource dynamics. Am Nat 166: 184–198. 16032573 10.1086/431285

[21] Vijverberg J, Vos M (2006) Predator-released compounds, ambient temperature and competitive exclusion among differently sized *Daphnia* species. Freshwater Biol 51: 756–767.

[22] Hoekman D (2010) Turning up the heat: Temperature influences the relative importance of top-down and bottom-up effects. Ecology 91: 2819–2825. 21058543 10.1890/10-0260.1

[23] Kratina P, Greig HG, Thompson PL, Carvalho-Pereira TSA Shurin JB (2012) Warming modifies trophic cascades and eutrophication in experimental freshwater communities. Ecology 93: 1421–1430. 22834382 10.1890/11-1595.1

[24] Halbach U (1970) Einfluss der Temperatur auf die Populationsdynamik des planktischen Rädertieres *Brachionus calyciflorus* . Oecologia 4: 176–207. 28309579 10.1007/BF00377100

[25] Tuda M, Schimada M (1995) Developmental schedules and persistence of experimental host-parasitoid systems at two different temperatures. Oecologia 103: 283–291. 28306821 10.1007/BF00328616

[26] Beisner BE, McCauley E, Wrona FJ (1996) Temperature-mediated dynamics of planktonic food chains: the effect of an invertebrate carnivore. Freshwater Biol 35: 219–232.

[27] Beisner BE, McCauley E, Wrona FJ (1997) The influence of temperature and food chain length on plankton predator-prey dynamics. Can J Fish Aquat Sci 54: 586–595.

[28] Beisner BE, McCauley E, Wrona FJ (1997) Predator-prey instability: individual-based mechanisms for population-level results. Funct Ecol 11: 112–120.

[29] Petchey OW, McPhearson PT, Casey TM, Morin PJ (1999) Environmental warming alters food web structure and ecosystem function. Nature 402: 69–72.

[30] Lundberg P, Ranta E, Kaitala V (2000) Species loss leads to community closure. Ecol Lett 3: 465–468.

[31] Oaten A, Murdoch WW (1975) Functional response and stability in predator-prey systems. Am Nat 106: 289–298.

[32] Abrams PA, Walter CJ (1996) Invulnerable prey and the paradox of enrichment. Ecology 77: 1125–1133.

[33] Case TJ (2000) An illustrated guide to theoretical ecology. Oxford University Press. 248 p.

[34] Altwegg R, Eng M, Caspersen S, Anholt BR (2006) Functional response and prey defence level in an experimental predator-prey system. Evol Ecol Res 8: 115–128.

[35] Mack TP, Smilowitz Z (1982) Using temperature-mediated functional response models to predict the impact of *Coleomegilla maculate* (DeGeer) adults and 3^rd^-instar larvae on green peach aphids. Environ Entomol 11: 46–52.

[36] Cave RD, Gaylor MJ (1989) Functional Response of *Telenomus reynoldsi* [Hym.: Scelionidae] at five constant temperatures and in an artificial plant arena. Biocontrol 34: 3–10.

[37] Flinn PW (1991) Temperature-dependent functional response of the parasitoid *Cephalonomia waterstoni* (Gahan) (Hymenoptera: Bethylidae) attacking rusty grain beetle larvae (Coleoptera: Cucujidae). Environ Entomol 20: 872–876.

[38] Menon A, Flinn PW, Dover BA (2002) Influence of temperature on the functional response of *Anisopteromalus calandrae* (Hymenoptera: Pteromalidae), a parasitoid of *Rhyzopertha dominica* (Coleoptera: Bostrichidae). J Stored Prod Res 38: 463–469.

[39] Xia J, Rabbinge R, Van der Werf W (2003) Multistage functional responses in a ladybeetle aphid system: scaling up from the laboratory to the field. Environ Entomol 32: 151–162.

[40] Huey RB, Kingsolver JG (1989) Evolution of thermal sensitivity of ectotherm performance. Trend Ecol Evol 4: 131–135. 10.1016/0169-5347(89)90211-5 21227334

[41] Martin TL, Huey RB (2008) Why “suboptimal” is optimal: Jensen's inequality and ectotherm thermal preferences. Am Nat 171: 102–118. 10.1086/527502 18271721

[42] Angilletta MJ (2009) Thermal adaptation: a theoretical and empirical synthesis. Oxford University Press.

[43] Dell AI, Pawar S, Savage VM (2011) Systematic variation in the temperature dependence of physiological and ecological traits. Proc Natl Acad Sci 108: 10591–6. 21606358 10.1073/pnas.1015178108 PMC3127911

[44] Guillard RRL, Lorenzen CJ (1972) Yellow-green algae with chlorophyllide c. J Phycol 8: 10–14.

[45] Brown JH, Gillooly JF, Allen AP, Savage VM, West GB (2004) Toward a metabolic theory of ecology. Ecology 85: 1771–1789.

[46] Sentis A, Hemptinne JL, Brodeur J (2012) Using functional response modeling to investigate the effect of temperature on predator feeding rate and energetic efficiency. Oecologia 169: 1117–1125. 22271203 10.1007/s00442-012-2255-6

[47] Sentis A, Hemptinne JL, Brodeur J (2013) Parsing handling time into its components: implications for responses to a temperature gradient. Ecology 94: 1675–1680. 24015511 10.1890/12-2107.1

[48] Fussmann GF, Weithoff G, Yoshida T (2005) A direct, experimental test of resource vs. consumer dependence. Ecology 86: 2924–2930.

[49] Peters RH (1984) Methods for the study of feeding, grazing and assimilation by zooplankton. In: Downing JA, Riegler FH (ed.), A Manual on Methods for the Assessment of Secondary Productivity in Freshwaters. Blackwell, 336–412 pp.

[50] Juliano SA, (1993) Nonlinear curve fitting: predation and functional response curves. In: Schreiner SM, Gurev-Itch J (ed.), Design and Analysis of Ecological Experiments. Chapman and Hall. 159–182 pp.

[51] Kratina P, Vos M, Bateman A, Anholt BR (2009) Functional responses modified by predator density. Oecologia 159: 425–433. 19034528 10.1007/s00442-008-1225-5

[52] Binzer A, Guill C, Brose U, Rall BC (2012) The dynamics of food chains under climate change and nutrient enrichment. Phil Trans R Soc B 1605: 2935–2944. 10.1098/rstb.2012.0230 PMC3479739 23007081

[53] Galkovskaja GA (1987) Planktonic rotifers and temperature. Hydrobiologia 147: 307–317.

[54] Jankowski T, Linvingstone DM, Bührer H, Forster R, Niederhauser P (2006) Consequence of the 2003 European heat wave for lake temperatue profiles, thermal stability and hypolimnetic oxygen depletion: Implications for a warmer world. Limnol Oceanogr 51: 815–819.

[55] Deutsch CA, Tewksbury JJ, Huey RB, Sheldon KS, Ghalambor CK, et al (2008) Impacts of climate warming on terrestrial ectotherms across latitude. Proc Natl Acad Sci 105: 6668–6672. 18458348 10.1073/pnas.0709472105 PMC2373333

