# Supplementary material for: Heated Relations: Temperature-Mediated Shifts in Consumption across Trophic Levels
Source: PLoS One. 2014 May 5;9(5):e95046. doi: 10.1371/journal.pone.0095046 (PMC4010407; doi:10.1371/journal.pone.0095046)
Supplement: Appendix S1 — (DOCX) [file pone.0095046.s001.docx]

**Appendix**

Table S1. Estimated parameters and confidence intervals for the different functional response models tested (Herb=herbivore data, Carn=carnivore data; Int=Integration; t=temperature [°C]; b=Hill exponent; a=attack rate; h=handling time; _Low/_High=lower/ higher limits of confidence intervals (95%); ad. r^2^=adjusted r2; bounded=estimate of the parameter reached the boundary value).

| Data | Int | T | b | a | a_Low | a_High | h | h_Low | h_High | ad.r2 |
| --- | --- | --- | --- | --- | --- | --- | --- | --- | --- | --- |
| Herb | No | 10 | 1 | 1.70E-03 | -6.71E-04 | 4.07E-03 | 3.50E-04 | -6.39E-04 | 1.34E-03 | 0.33 |
| Herb | No | 15 | 1 | 7.00E-03 | -1.12E-03 | 1.51E-02 | 9.99E-04 | 5.86E-04 | 1.41E-03 | 0.40 |
| Herb | No | 20 | 1 | 1.23E-02 | 3.32E-03 | 2.13E-02 | 4.84E-04 | 3.45E-04 | 6.24E-04 | 0.63 |
| Herb | No | 25 | 1 | 2.47E-02 | 1.06E-02 | 3.88E-02 | 4.06E-04 | 3.37E-04 | 4.75E-04 | 0.73 |
| Herb | No | 10 | 2 | 6.34E-09 | bounded | bounded | 7.87E-04 | 4.39E-04 | 1.14E-03 | 0.35 |
| Herb | No | 15 | 2 | 7.13E-08 | -3.98E-08 | 1.82E-07 | 1.19E-03 | 8.92E-04 | 1.48E-03 | 0.41 |
| Herb | No | 20 | 2 | 1.75E-07 | -3.43E-09 | 3.54E-07 | 6.15E-04 | 5.14E-04 | 7.17E-04 | 0.60 |
| Herb | No | 25 | 2 | 3.24E-07 | 8.63E-08 | 5.62E-07 | 4.67E-04 | 4.15E-04 | 5.19E-04 | 0.73 |
| Herb | Yes | 10 | 2 | 6.36E-9 | bounded | bounded | 7.87E-4 | 4.39E-4 | 1.14E-3 | 0.35 |
| Herb | Yes | 15 | 2 | 7.16E-8 | -4.01E-8 | 1.83E-7 | 1.19E-3 | 8.92E-4 | 1.48E-3 | 0.41 |
| Herb | Yes | 20 | 2 | 1.76E-7 | -3.89E-9 | 3.55E-7 | 6.15E-4 | 5.13E-4 | 7.17E-4 | 0.60 |
| Herb | Yes | 25 | 2 | 3.28E-7 | 8.63E-8 | 5.70E-7 | 4.67E-4 | 4.15E-4 | 5.19E-4 | 0.73 |
| Carn | No | 10 | 1 | 1.39E-01 | -2.00E-03 | 2.81E-01 | 5.68E-01 | 8.50E-02 | 1.05E+00 | 0.15 |
| Carn | No | 15 | 1 | 1.72E-01 | -1.00E-03 | 3.46E-01 | 6.03E-01 | 2.04E-01 | 1.00E+00 | 0.15 |
| Carn | No | 20 | 1 | 4.52E-01 | 5.30E-02 | 8.51E-01 | 5.34E-01 | 3.77E-01 | 6.92E-01 | 0.12 |
| Carn | No | 25 | 1 | 2.54E-01 | 1.34E-01 | 3.75E-01 | 2.55E-01 | 1.55E-01 | 3.55E-01 | 0.33 |
| Carn | No | 10 | 2 | 3.60E-02 | -1.20E-02 | 8.30E-02 | 8.95E-01 | 6.16E-01 | 1.18E+00 | 0.14 |
| Carn | No | 15 | 2 | 2.90E-02 | -3.00E-03 | 6.10E-02 | 8.00E-01 | 5.68E-01 | 1.03E+00 | 0.17 |
| Carn | No | 20 | 2 | 1.22E-01 | -1.10E-02 | 2.56E-01 | 6.38E-01 | 5.42E-01 | 7.34E-01 | 0.13 |
| Carn | No | 25 | 2 | 3.89E-02 | 1.70E-02 | 6.10E-02 | 3.76E-01 | 3.17E-01 | 4.35E-01 | 0.34 |
| Carn | Yes | 10 | 2 | 4.65E-02 | -2.38E-02 | 1.17E-01 | 8.95E-01 | 6.12E-01 | 1.18E+00 | 0.14 |
| Carn | Yes | 15 | 2 | 4.14E-02 | -1.19E-02 | 9.47E-02 | 8.13E-01 | 5.79E-01 | 1.05E+00 | 0.17 |
| Carn | Yes | 20 | 2 | 3.14E-01 | -1.95E-01 | 8.23E-01 | 6.53E-01 | 5.59E-01 | 7.47E-01 | 0.13 |
| Carn | Yes | 25 | 2 | 7.24E-02 | 1.91E-02 | 1.26E-01 | 3.85E-01 | 3.25E-01 | 4.45E-01 | 0.33 |
